# A novel variant in *NBAS* identified from an infant with fever-triggered recurrent acute liver failure disrupts the function of the gene

**DOI:** 10.1038/s41439-023-00241-0

**Published:** 2023-04-13

**Authors:** Juhua Ji, Mingming Yang, JunJun Jia, Qi Wu, Ruochen Cong, Hengxiang Cui, Baofeng Zhu, Xin Chu

**Affiliations:** 1 Department of Pediatrics, The Second Affiliated Hospital of Nantong University, 226001 Nantong, Jiangsu China; 2 Department of Biochemistry and Molecular Biology, School of Medicine, Nantong University, 226001 Nantong, Jiangsu China; 3 Qinshen Traditional Chinese Medicine (TCM) Outpatient Department, 20052 Shanghai, China; 4 Department of Emergency, The Second Affiliated Hospital of Nantong University, 226001 Nantong, Jiangsu China; 5 Department of Radiology, The Second Affiliated Hospital of Nantong University, 226001 Nantong, Jiangsu China; 6 Medical Research Center, The Second Affiliated Hospital of Nantong University, 226001 Nantong, Jiangsu China

**Keywords:** Genetics, Mutation

## Abstract

Mutations in the neuroblastoma amplified sequence (*NBAS*) gene correlate with infantile acute liver failure (ALF). Herein, we identified a novel *NBAS* mutation in a female infant diagnosed with recurrent ALF. Whole-exome and Sanger sequencing revealed that the proband carried a compound heterozygous mutation (c.938_939delGC and c.1342 T > C in *NBAS*). *NBAS* c.938_939delGC was presumed to encode a truncated protein without normal function, whereas *NBAS* c.1342 T > C encoded NBAS harboring the conserved Cys448 residue mutated to Arg448 (p.C448R). The proportion of CD4 + T cells decreased in the patient’s peripheral CD45 + cells, whereas that of CD8 + T cells increased. Moreover, upon transfecting the same amount of DNA expression vector (ectopic expression) encoding wild-type NBAS and p.C448R NBAS, the group transfected with the p.C448R NBAS-expressing vector expressed less *NBAS* mRNA and protein. Furthermore, ectopic expression of the same amount of p.C448R NBAS protein as the wild-type resulted in more intracellular reactive oxygen species and the induction of apoptosis and expression of marker proteins correlating with endoplasmic reticulum stress in more cultured cells. This study indicated that p.C448R NBAS has a function different from that of wild-type NBAS and that the p.C448R NBAS mutation potentially affects T-cell function and correlates with ALF.

## Liver disease: Mutation linked with acute liver failure in infants

Researchers in China have identified a new mutation in the gene *NBAS* that is associated with acute liver failure (ALF) in infants. Juhua Ji at Nantong University and colleagues found the mutation in a 26-month-old girl who had a high fever, vomiting, abdominal distention, and poor food intake. After diagnosing ALF through blood tests, the team sequenced the infant’s DNA to find potential causative mutations. There were two mutations in the *NBAS* gene, which has been previously linked with ALF. Further analysis showed that one of the mutations induces oxidative stress and changes the ratio of types of T-helper cells, which the researchers suggest leads to ALF. These findings not only identify a novel pathogenic *NBAS* mutation but also offer a tool to investigate links between *NBAS*, immune cells, and ALF progression.

## Introduction

Acute liver failure (ALF) is a life-threatening event in infancy and childhood that can result from infectious diseases, inherited metabolic diseases, toxin exposure, autoimmune diseases, shock, and other causes^1^. The cause of this disease remains unknown in ~50% of cases, although almost 25% of ALF cases result from congenital metabolism^2–4^. Regarding genetic factors, researchers have identified several mutations related to ALF in genes such as neuroblastoma amplified sequence (*NBAS*)^2–6^, *SCYL1*^3^, E3 ubiquitin-protein ligase^7^, perforin^8^, *LARS*^9^, *ATP7B*^10^, *RINT1*^11^, and mitochondrial *TRMU* genes^12^. Among these genes, *NBAS* is the most widely studied, and the genotype–phenotype correlation has recently been deciphered^2^. Because the progression of ALF with an *NBAS* mutation can be effectively ameliorated by early antipyretic therapy and the induction of anabolism, it is crucial to differentially diagnose *NBAS* mutation-based disease. In this study, we identified the novel compound heterozygous missense mutations c.938_939delGC and c.1342 T > C in *NBAS* in a Chinese female with recurrent episodes of ALF. Moreover, we discovered that the patient had different CD4^+^ or CD8^+^ T-cell populations than normal children and that p.C448R NBAS protein has a different function than that of the wild-type NBAS protein. These findings suggest that the c.1342 T > C mutation in *NBAS* might be a pathogenic variant.

## Materials and methods

### Cell culture and DNA transfection

HEK293T (crl-11268) and Jurkat (clone E6-1) cells were obtained from ATCC (Rockville, MD, USA) HEK293T cells were maintained in high glucose-containing DMEM (Life Technologies, USA) supplemented with 10% fetal bovine serum (FBS) and 1% antibiotics (penicillin‒streptomycin) at 37 °C with 5% CO_2_. Jurkat cells were cultured with advanced RPMI 1640 (Gibco) supplemented with 10% fetal bovine serum, 10 mM HEPES, and 1% antibiotics (penicillin‒streptomycin) at 37 °C with 5% CO_2_.

DNA transfection was conducted using Lipofectamine LTX Reagent (#15338100, Thermo Fisher Scientific). Transfection was strictly conducted following the manufacturer’s instructions. In addition, 24 h post-transfection, the medium in each treatment was changed. Furthermore, after another 24 h of incubation at 37 °C in a CO_2_ incubator, the cells in each group were used to conduct the reactive oxygen assay, apoptosis analysis, and immunoblot assay.

### Reactive oxygen species (ROS) detection using DCFDA

A DCFDA cellular ROS assay kit (D399, Invitrogen) was used to detect cellular reactive oxygen species (ROS) with a minor change in the product instructions. Briefly, Jurkat cells were collected by centrifugation at 300 × *g* before being washed with buffer and stained with DCFDA for 40 min. Then, in each wash (total of three washes) using the kit’s buffer, the cells were collected by centrifugation at 300 × *g*. Finally, 40–50 μl of wash buffer was added to the cell pellets to obtain the cell suspension. Then, the cell suspension was seeded into a 24-well plate and analyzed with a fluorescence microscope. No fewer than 18 pictures in each group were taken randomly for evaluation.

### Whole-exome sequencing

Peripheral blood DNA from the proband was used to conduct whole-exome sequencing by Mingma Biotechnology (https://www.mingmatechs.com/) using the HiSeq X10 System (Illumina Inc., San Diego, CA, USA). Raw reads were assigned following the human genome GRCh37 and distinguished using an in-house method that included Burrows‒Wheeler Aligner mapping and the FreeBayes variant calling protocol. During the analysis, mapped reads were filtered by comparison with in-house and public databases. Potential pathogenic gene variations correlating to ALF or those containing autosomal recessive mutations were listed as candidate genes.

### Sanger direct sequencing

To verify the two variants in exons 11 and 15 of the *NBAS* gene obtained by whole-exome sequencing, Sanger sequencing of the polymerase chain reaction (PCR) products from PCR amplification was performed using genomic DNA from the patient and her parents as templates. PCR amplification was conducted using the primers for *NBAS* exon 11 (forward 5ʹ-GAGAAGAGCTTGCGGTGGAT-3ʹ, reverse 5ʹ-CCAGTGTCTTCGGTACCTGC-3ʹ) and exon 15 (forward 5ʹ-GAGAAGAGCTTGCGGTGGAT-3ʹ, reverse 5ʹ-CCAGTGTCTTCGGTACCTGC-3ʹ). PCR products were sequenced by Biosun Biotechnology Shanghai Co. Ltd. (Shanghai, China).

### Analysis of peripheral lymphocytes

During emergency treatment, the peripheral blood of the patient was collected three times every 3 days; in addition, the peripheral blood of three normal children of the same age and gender were collected on the same day as the patient. For each timepoint for analysis, the peripheral blood samples included one specimen from the patient and one from a normal child. The isolated peripheral blood lymphocytes from the patient and children without ALF were stained with fluorescently labeled anti-CD45, CD3, CD4, and CD8 antibodies before being loaded into the flow cytometry assay, following the instructions of the CD45-FITC/CD4-PE/CD8-ECD/CD3-PC5 antibody cocktail (6607013, Beckman).

### NBAS gene synthesis and vector construction

The human *NBAS* cDNA ORF clone (7116 bp) was synthesized by Jiangsu Dongxuan Gene Technology Co. Ltd. Full-length *NBAS* cDNA was obtained by PCR using LA Taq® DNA Polymerase. The primers used in this study were as follows: forward primer 5ʹ-GGGGATATCATGGCGGCCCCCGAGTCAG-3ʹ and reverse primer 5ʹ-TGG CGGCCGCCTACTTGTCATCGTCGTCCTTGTAGTCCACCCAGTGCTGTGCTGC-3ʹ. The underlined letters represent the cleavage sites of the restriction endonucleases used for vector construction. After digestion by the endonucleases EcoRV and NotI, the PCR product was inserted into the pcDNA3.1(+) vector with T4 ligase to form pCDNA3.1-NBAS-Flag. Using the pcDNA3.1-NBAS-Flag vector as a template, PCR amplification was conducted with the following primer pairs: NBAS-C448R forward primer, 5ʹ-TTTAAGTTTGGAGCGTAGATTAAACTTGCCCCCAAACGATCTCGTTTGGAG-3ʹ; NBAS-C448R reverse primer, 5ʹ-CACGCTCCAAACTTAAAAATCCCCCATCATGGGTAGCAGTGAC-3ʹ. The PCR products were digested with DpnI before recombination by GeneArt™ Gibson Assembly HiFi (Invitrogen) to obtain pcDNA3.1-NBAS-C448R-Flag.

### RNA extraction and quantitative (real-time) PCR

As described in a previous study^13^, total RNA was extracted from the cells using TRIzol reagent (Invitrogen, Carlsbad, CA, USA). Total RNA was then used to conduct reverse transcription using PrimeScript RT Reagent Kits (Takara, Kusatsu, Japan) following cDNA. cDNA was amplified in triplicate via qRT‒PCR using the CFX Connect Real-Time PCR Detection System (Bio-Rad, CA, USA) and SYBR Premix Ex Taq (Takara Bio), with β-actin as an internal control. The qRT‒PCR cycling was designed as follows: denaturation at 95 °C for 3 min, 95 °C for 35 s, and annealing at 60 °C for 35 s over 40 cycles. The 2^−ΔΔCT^ method was used to calculate the relative expression.

### Western blot analysis

Western blotting was conducted as previously described^13^. Briefly, the cells were washed with PBS and lysed with RIPA buffer (#89901; Thermo Scientific) containing a protease inhibitor cocktail (#78420). The lysate was centrifuged at 13,000 × *g* for 10 min at 4 °C before the supernatant was collected and the protein concentration was measured. Furthermore, 20–80 µg of protein was separated by sodium dodecyl sulfate‒polyacrylamide gel electrophoresis before being electrotransferred to a nitrocellulose membrane. The membrane was blocked with 5% nonfat milk in TBST before incubation with primary antibodies. The antibodies used were anti-ATF4 (ab184909, Abcam), anti-XBP1 (ab37152, Abcam), anti-CHOP (#5554, CST), anti-BiP (#3177, CST), anti-FLAG tag (#14793, CST), anti-NBAS (#PA5–49534, Thermo Fisher Scientific) and anti-β-actin (#4967, CST). Specific peroxidase-conjugated secondary antibodies and an enhanced chemiluminescence kit (Pierce; Thermo Fisher Scientific, Waltham, MA, USA) were used for enzyme-catalyzed reactions to amplify the protein signals. Enhanced chemiluminescence signals were analyzed using ImageJ software (v1.50i) (US, Bethesda, MD, USA).

### Cell apoptosis assay

Cell apoptosis was determined using a PE Annexin V Apoptosis Detection Kit (BD Biosciences, USA) in three independent experiments^14^. Briefly, Jurkat cells from each treatment group were collected by centrifugation. The cells were then resuspended at 1 × 10^6^ cells/mL at room temperature. Each 100 μL of cell suspension was incubated with 7-AAD and annexin V-FITC at room temperature for 15 min following the manufacturer’s instructions. Finally, the forward cell suspension was added to another 400 μL of binding buffer before analysis using a flow cytometer (CytoFLEX, Beckman, USA). The percentage of apoptotic cells for each treatment was analyzed using the FlowJo application (V10).

## Results

During emergency treatment, a 26-month-old sick female infant (height, 80.5 cm −2 SD height standard deviation score; weight, 9 kg −3 SD weight standard deviation score) was sent to our hospital for treatment for a fever accompanied by a thermal spike at 40 °C and vomiting. The patient had slight abdominal distension, poor food intake, and drowsiness and was negative for the Babinski, Brudzinski, and Kernig signs. From seven months old, the patient had been repeatedly hospitalized in our department because of fever and abnormal liver function, with the highest serum level of glutamic pyruvic transaminase at 1629 U/L. Pathological slices showed cloudy swelling in the liver cells.

Blood tests were performed upon hospital admission to assess liver and kidney function. The findings revealed that the levels of glutamic pyruvic transaminase, aspartate aminotransferase, glutamyltranspeptidase, total bilirubin, direct bilirubin, total bile acid, and adenosine deaminase were higher than the reference values, whereas the levels of alkaline phosphatase and indirect bilirubin were lower than the reference values in the serum (Table 1). In the evaluation of kidney function, the creatinine level (27.10 μmol/L) was lower than the reference range of 50–100 μmol/L, whereas the uric acid level (461.20 μmol/L) was higher than the reference range of 208–506 μmol/L. The ammonia level in the blood was 57.50 μmol/L, which was much higher than the reference value of 8–35 μmol/L. In addition, the alpha-fetoprotein and cytomegalovirus IgM antibody levels in the serum of the patient were higher than the reference values of 0–10 g/ml and 0–12 AU/ml, with levels reaching 16.85 g/ml and 12.922 AU/ml, respectively. Other autoantibodies related to hepatitis in the serum were also tested. The anti-AMA-M2 and anti-M2–3E antibodies tested positive in the patient’s serum (Supplementary Table S1). Regarding overall immune function, the levels of immunoglobulin κ-light chain, immunoglobulin λ-light chain, and immunoglobulin G were 5.40 g/L, 2.54 g/L, and 22.75 g/L, respectively, all of which were higher than the reference ranges of 2–4.4 g/L, 1.1–2.4 g/L, and 6.8–14.45 g/L, respectively.

**Table 1:** Blood test results for assessing liver and kidney function.

Parameters	Feb 25th values	Feb 27th values	Reference
Glutamic pyruvic transaminase	7011 U/L	2875 U/L	5–40 U/L
Aspartate aminotransferase	8103 U/L	901 U/L	5–35 U/L
Glutamyltranspeptidase	45 U/L	74 U/L	7–45 U/L
Alkaline phosphatase	274 KU/L	314 KU/L	35–135 KU/L
Total bilirubin	42.1 μmol/L	54.4 μmol/L	1.70–17.1
Direct bilirubin	37.9 μmol/L	52.5 μmol/L	0–7.0 μmol/L
Indirect bilirubin	4.2 μmol/L	1.9 μmol/L	3.0–16.0 μmol/L
Total bile acid	374 μmol/L	426.90 μmol/L	0–20 μmol/L
Adenosine deaminase	45.9 U/L	41.6 U/L	5.0–25.0 U/L

Note that Feb 25th and Feb 27th values were tested on February 25th and 27th, respectively.

In addition to liver function evaluation, we also assessed the blood coagulation of the patient. The findings revealed that the D-dimer level, prothrombin time, fibrinogen-degradation products, and international normalized ratio (INR = 2.77), which is a mean value of risk stratification among patients with liver disease, were higher than the reference ranges (Table 2). As the main clinical presentation of this patient comprised hyperammonemia, hepatic dysfunction, abnormal liver biochemical values, and coagulopathy, we diagnosed this patient with ALF.

**Table 2:** Blood test results for assessing coagulation-related parameters.

Parameters	Values	Reference
Fibrinogen	2.60 g/L	2.00–4.00 g/L
Thrombin time	20.1 s	14.0–21.0 s
Prothrombin time	29.8 s	10.0–16.0 s
International normalized ratio	2.77	0.82–1.50
Partial thromboplastin time	31.4 s	20.0–40.0 s
D-dimer	9,672.0 μg / L	0–1,000 μg / L
Anti-thrombin III	394.4%	70.0–140.0%
Fibrinogen-degradation products	18.23 μg/mL	0.00–5.00 μg/mL

**Fig. 1:**
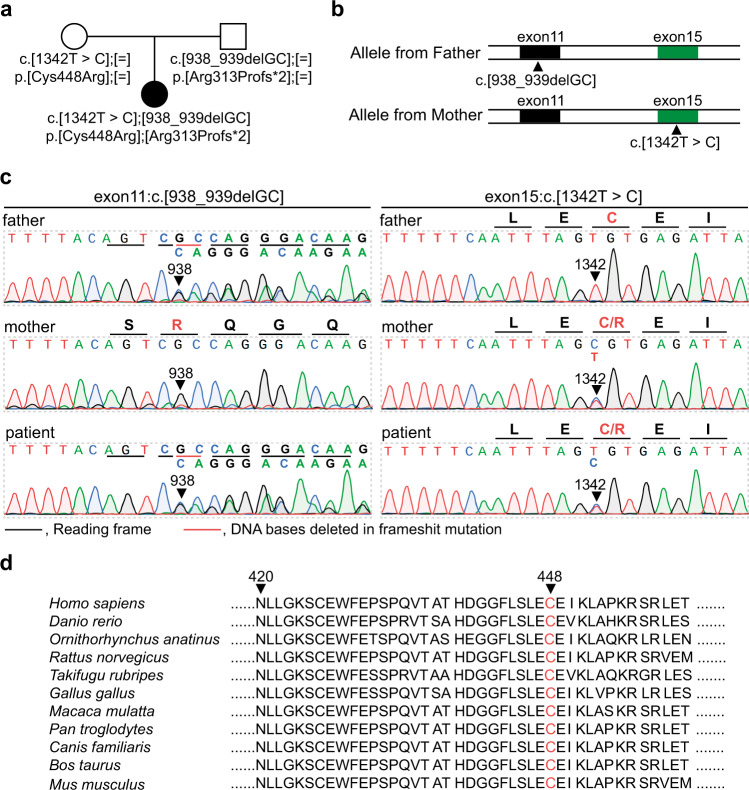
Identification of compound heterozygous mutations in the NBAS gene in the patient and her family. **a**, **b** Family pedigree. The patient’s mother carried the c.1342 T > C (p.Cys448Arg) missense mutation in one *NBAS* allele, whereas the patient’s father carried the c.938_939delGC frameshift mutation. **c** Sanger sequence analysis confirmed the segregation of two *NBAS* mutations: NBAS c.938_939delGC and c.1342 T > C (p.Cys448Arg) in this family. **d** The c.1342 T > C (p.Cys448Arg) mutation affects evolutionarily conserved amino acid residues in multiple species.

We further investigated whether ALF in the patient was caused by a genetic mutation. Using whole-exome sequencing, we found that the mutation of *NBAS*, which is associated with recurrent acute liver failure when mutated^3,5^, most likely was a potential pathogenic cause in the patient. Further Sanger sequencing was used to confirm the results from whole-exome sequencing. The Sanger sequencing results showed that the mother of the proband carried the heterozygous missense mutation c.1342 T > C in exon 15 of the *NBAS* gene, and the father of the proband carried the heterozygous frameshift insertion mutation c.938_939delGC in exon 11 of the *NBAS* gene (Fig. 1a–c). Moreover, the patient’s *NBAS* gene contained the heterozygous frameshift insertion mutation c.938_939delGC in the *NBAS* gene from the paternal allele and the missense mutation c.1342 T > C from the maternal allele (Fig. 1a–c). Thus, the patient carried a compound heterozygous mutation in the *NBAS* gene. In this compound heterozygous mutation, the frameshift insertion mutation c.938_939delGC from the paternal allele would cause translation failure of functional NBAS proteins, whereas the missense mutation c.1342 T > C from the maternal allele would cause the NBAS protein to have a p.C448R mutation localized at the edge of the β-propeller domain in the NBAS protein^2^. We further revealed that the p.C448R mutation in the NBAS protein is highly conserved in multiple species (Fig. 1d), indicating that this mutation might correlate to the phenotype of the patient.

**Fig. 2:**
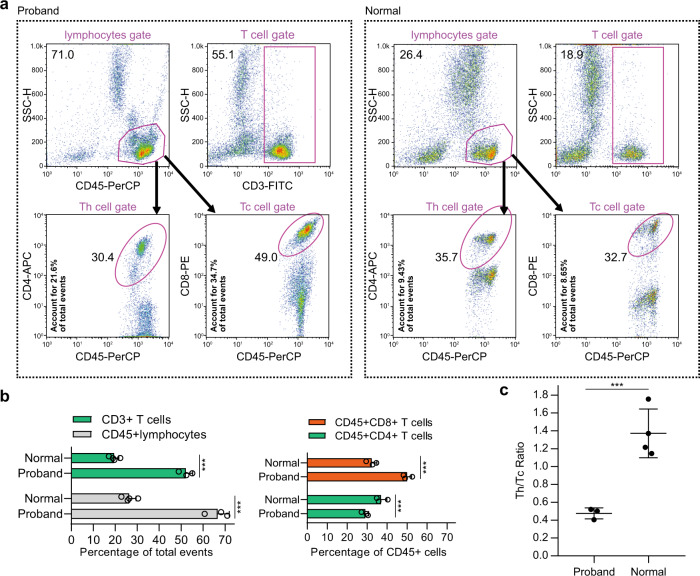
Analysis of peripheral lymphocytes by flow cytometry. **a** Representative flow cytometry profile based on the surface markers CD3, CD45, CD4, and CD8 in the peripheral lymphocytes of the patient and children without ALF. The cells were identified using CD45 and CD3 gates. The CD4 and CD8 T cells were gated out from the CD45-positive T-cell gate in each cell sample. **b** Columns represent the flow cytometry analysis of markers for populations enriched in CD45^+^ lymphocytes, CD3^+^ T lymphocytes, Th (CD4^+^CD45^+^), or Tc (CD8^+^CD45^+^) in the peripheral lymphocytes of the patient (three tests) and three children without ALF. **c** Scatter dot plot of the flow cytometry analysis of markers for populations enriched in Th (CD3^+^CD4^+^CD45^+^) or Tc (CD3^+^CD8^+^CD45^+^) cells in the peripheral lymphocytes of the patient (three tests) and four children without ALF. Bars indicate the mean values. ***P < 0.001 from two-tailed Student’s *t* tests.

Because T-lymphocyte subsets could be important in the pathogenesis of ALF^9,15^, we further analyzed changes in the peripheral T lymphocytes of the proband using flow cytometry. The results showed that the subtypes of lymphocytes carrying antigens for CD45^+^ and CD3^+^ were higher in the proband than in children without ALF (Fig. 2a, b). In CD45^+^ cells, the proportion of peripheral CD4^+^ T cells was decreased, but CD8 + T cells increased in the patient compared to the proportion in children without ALF (Fig. 2a, b). The ratio of CD3^+^CD4^+^ Th cells to CD3^+^CD8^+^ cytotoxic T lymphocytes in the proband was significantly lower than that in children without ALF (Fig. 2c).

**Fig. 3:**
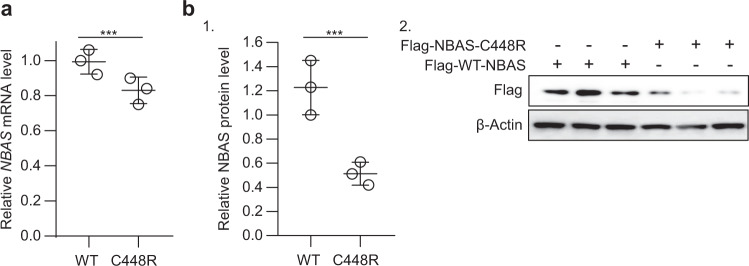
Ectopic expression of *NBAS* C448R led to less mRNA transcription and protein expression than that of wild-type *NBAS*. **a**, **b** After transfecting the same amount of pcDNA3.1-NBAS-Flag and pcDNA3.1-NBAS-C448R-Flag (300 ng/well for each well of 24-well plate) into HEK293T cells, the expression of *NBAS* mRNA at 16 h after transfection (**a**) and Flag-tagged NBAS protein at 24 h after transfection (**b**) were determined by qRT‒PCR and western blot analysis, respectively, with quantification of immune blot results shown (B1). All data are presented as the mean ± SD. ***P < 0.001 from two-tailed Student’s *t* tests.

**Fig. 4:**
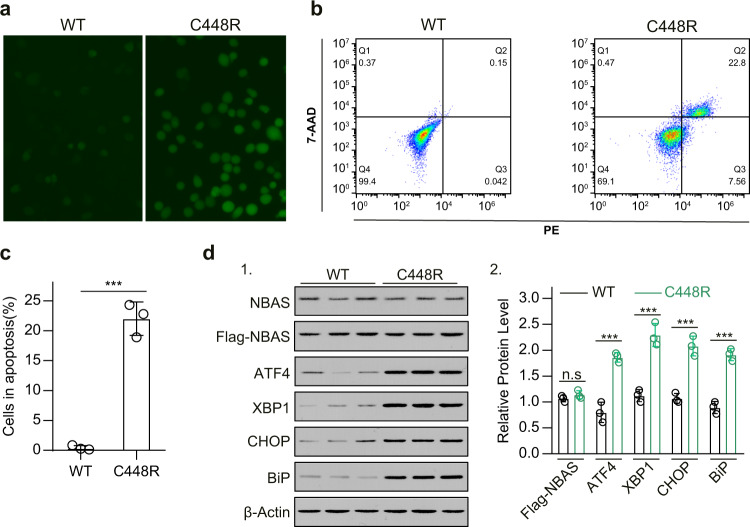
Ectopic expression of *NBAS* C448R induced greater intracellular reactive oxygen species stress and endoplasmic reticulum stress than that of wild-type *NBAS* in vitro. **a** Forced expression of NBAS C448R increased the reactive oxygen species in Jurkat cells detected by the H2DCFDA probe (D399, Invitrogen) following the product instructions. Jurkat cells in each group were incubated with 7-AAD and annexin V-FITC before being analyzed in a flow cytometer three times (**b**). The percentages of apoptotic cells were quantified, as shown in the right column (**c**). **d** Ectopic expression of NBAS C448R regulated the expression of the indicated ER stress-related proteins in Jurkat cells, as detected by western blot analysis (F1, left panel), with quantification of related protein expression shown in F2 (right panel). In each experiment, 750 ng NBAS-C448R-Flag or 300 ng NBAS-C448R-Flag was transfected into each well of a 24-well plate. Moreover, cells were used for assays at 24–30 h after transfection. Experiments were performed in triplicate and repeated three times with similar results. All data are presented as the mean ± SD. ***P < 0.001 from two-tailed Student’s *t* tests.

To uncover the potential mechanism by which this mutation contributes to ALF progression, we investigated whether the mutation affected *NBAS* mRNA and protein expression. We discovered that when the same amount of wild-type or C448R-mutated *NBAS* vectors were transfected, the mRNA expression (transcription from endogenous and vector NBAS) (Fig. 3a) and mutated NBAS protein expression were significantly lower in the group transfected with the *NBAS* C448R construct (Fig. 3b). This indicated that the c.1342 T > C mutation in the *NBAS* gene may attenuate *NBAS* mRNA expression, which in turn decreases NBAS protein expression. We also found that ectopic expression of NBAS C448R at nearly the same level as wild-type NBAS increased reactive oxygen species in Jurkat cells (an immortalized T-lymphocyte cell line) (Fig. 4a). Moreover, ectopic expression of NBAS C448R at the same level of wild-type NBAS induced apoptosis in Jurkat cells (Fig. 4b, c). These results support that the p.C448R NBAS mutation does not affect endogenous *NBAS* gene-encoded protein expression (Fig. 4d), and p.C448R NBAS protein has a different function than the NBAS protein. Furthermore, we also discovered that the p.C448R NBAS protein might induce oxidative stress, which might change the survival of certain T-helping cell populations (Figs. 2 and 4a–d). *NBAS* regulates the stability of ER-associated transcripts, and the ER unfolded protein response^16^; thus, we hypothesized that p.C448R NBAS protein could induce ER stress. Western blotting revealed that overexpression of NBAS C448R significantly increased the expression of ER stress-related genes, including ATF4, XBP1, CHOP, and BIP (Fig. 4d)^17^.

Collectively, these preliminary results revealed that the c.1342 T > C mutation in the *NBAS* gene identified from the novel compound heterozygous mutation is highly conserved in multiple species and may affect the expression of mutated *NBAS* mRNA, which changes the mutated NBAS protein expression (Fig. 3). However, the NBAS C448R protein has a different function than wild-type NBAS and can induce oxidative stress and endoplasmic reticulum stress, which might affect the survival of certain T-cell populations (Figs. 2 and 4a–d).

## Discussion

Initially identified as coamplified with the *MYCN* gene in neuroblastoma cells, *NBAS* is believed to play an important role in Golgi-to-ER transport^18^. Harmful mutations in *NBAS* were originally related to a high prevalence of hereditary short stature syndrome^19^. Subsequently, growing evidence has supported the notion that *NBAS* mutations are associated with recurrent acute liver failure^3,5,20^. In this study, we identified the C448R mutation in NBAS as a novel compound heterozygous variation that is highly conserved in multiple species by combining whole-exome sequencing with Sanger sequencing using the proband’s genome from peripheral leukocytes.

*NBAS* is believed to be the second most frequently mutated gene in human hemophagocytic lymphohistiocytosis^21^, which is a life-threatening disease characterized by unbridled activation of cytotoxic T lymphocytes resulting in hypercytokinemia and immune-mediated injury of multiple organ systems. Mutations in the *NBAS* gene have previously been linked to immunologic aspects^21–23^, with general symptoms including decreased immunoglobulin G levels, CD56^+^ NK, and CD19^+^ naive B cells^22,24^. Our patient, who exhibited no decrease in CD19 B cells in peripheral blood (Supplementary Table S2), displayed normal IgG levels as in another study^20^; however, our result supported that cytotoxic T cells (CD45^+^CD8^+^ T cells) in the patient were higher than those in normal control children (Fig. 2). From this, we deduced that the NBAS C448R mutation might be associated with cytotoxic T-cell function, although a few studies reported that NBAS affected NK cells^20,22,23^. In addition, during the determination of autoantibodies related to hepatitis, anti-AMA-M2 and anti-M2–3E antibodies were found in the patient’s serum (Supplementary Table S1). Anti-mitochondrial M2 antibody, which is specific to primary biliary cirrhosis (PBC), is also found in some patients with autoimmune hepatitis (AIH)^25^. In addition, 4 of 10 patients who underwent liver biopsy and were diagnosed with AIH had mitochondrial M2 antibodies. Furthermore, our results help to enrich the understanding of the function of the *NBAS* gene in immune cells.

We also revealed that the *NBAS* c.1342 T > C mutation reduced the expression of *NBAS* mRNA and protein (Fig. 3a, b), which indicated that the c.1342 T > C variation was a loss-of-function mutation. Moreover, forced expression of p.C448R NBAS protein induced more expression of marker proteins correlating to endoplasmic reticulum stress in cultured cells (Fig. 4b–d), which was consistent with the finding that the *NBAS* mutant significantly increased the expression of genes involved in the ER stress response in fibroblasts^5^. This demonstrated that the variant c.1342 T > C in the *NBAS* gene not only affected gene expression but encoded a mutated *NBAS* with a different function than wild-type *NBAS*.

In summary, in a 26-month-old sick female infant, we identified the novel compound heterozygous missense mutations c.938_939delGC and c.1342 T > C in the *NBAS* gene and preliminarily suggest that the c.1342 T > C variation in *NBAS* may decrease the production of *NBAS* mRNA and functional *NBAS* proteins in an in vitro culture model. We also found that in the peripheral CD45-positive cells of the patient, the proportion of cytotoxic CD8 + T cells increased. Using an in vitro model, we revealed that the mutated C448R *NBAS* protein had a different function than wild-type NBAS. Further studies are needed to determine how *NBAS* affects immune cell function and thus contributes to the pathological progression of ALF.

### Supplementary information

Table S1

Table S2

## Data Availability

The datasets analyzed in the current study are available from the corresponding author upon reasonable request.
