# Supplementary material for: A novel variant in NBAS identified from an infant with fever-triggered recurrent acute liver failure disrupts the function of the gene
Source: Hum Genome Var. 2023 Apr 13;10:13. doi: 10.1038/s41439-023-00241-0 (PMC10102179; doi:10.1038/s41439-023-00241-0)
Supplement: Supplementary file 1 — Table S1 [file 41439_2023_241_MOESM1_ESM.docx]

**Table S1. Patient serum autoantibodies related to autoimmune hepatitis**

| **Parameters** | Reactivity |
| --- | --- |
| Anti-AMA-M2 antibody | + |
| Anti-M2-3E antibody | -/+ |
| Anti-Sp100antibody | - |
| Anti-PMLantibody | - |
| Anti-LKM-1antibody | - |
| Anti-LC-1 antibody | - |
| Anti-SLA/LP antibody | - |
| Anti-Ro52 antibody | + |

+, positive reaction; -, negative reaction; -/+, weakly positive reaction
