# Supplementary material for: A novel variant in NBAS identified from an infant with fever-triggered recurrent acute liver failure disrupts the function of the gene
Source: Hum Genome Var. 2023 Apr 13;10:13. doi: 10.1038/s41439-023-00241-0 (PMC10102179; doi:10.1038/s41439-023-00241-0)
Supplement: Supplementary file 2 — Table S2 [file 41439_2023_241_MOESM2_ESM.docx]

**Table S2. Peripheral lymphocyte analysis of the patient.**

| **Parameters** | **Reactivity (%)** | **Reference (%)** |
| --- | --- | --- |
| CD3^+^ cells | 77 | 62­–77 |
| CD4^+^ T cells | 27 | 26–41 |
| CD8^+^ T cells | 49 | 19–30 |
| CD4^+^CD8^+^ T cells | 0.55 | 1.2–2.9 |
| CD16^+^CD56^+^ cells | 56.9 | 10–22 |
| CD19^+^ B cells | 14 | 7–14 |
